# Disorder-induced topological phase transitions in two-dimensional spin-orbit coupled superconductors

**DOI:** 10.1038/srep39188

**Published:** 2016-12-19

**Authors:** Wei Qin, Di Xiao, Kai Chang, Shun-Qing Shen, Zhenyu Zhang

**Affiliations:** 1 International Center for Quantum Design of Functional Materials (ICQD), Hefei National Laboratory for Physical Sciences at Microscale (HFNL), and Synergetic Innovation Center of Quantum Information and Quantum Physics, University of Science and Technology of China, Hefei, Anhui, 230026, China; 2 Department of Physics, Carnegie Mellon University, Pittsburgh, Pennsylvania 15213, USA; 3 SKLSM, Institute of Semiconductors, Chinese Academy of Sciences, P. O. Box 912, Beijing 100083, China; 4 Department of Physics, The University of Hong Kong, Pokfulam Road, Hong Kong, China

## Abstract

Normal superconductors with Rashba spin-orbit coupling have been explored as candidate systems of topological superconductors. Here we present a comparative theoretical study of the effects of different types of disorder on the topological phases of two-dimensional Rashba spin-orbit coupled superconductors. First, we show that a topologically trivial superconductor can be driven into a chiral topological superconductor upon diluted doping of isolated magnetic disorder, which close and reopen the quasiparticle gap of the paired electrons in a nontrivial manner. Secondly, the superconducting nature of a topological superconductor is found to be robust against Anderson disorder, but the topological nature is not, converting the system into a topologically trivial state even in the weak scattering limit. These topological phase transitions are distinctly characterized by variations in the topological invariant. We discuss the central findings in connection with existing experiments, and provide new schemes towards eventual realization of topological superconductors.

Topological superconductors (TSCs)^1^ have been intensively explored recently as candidate systems for realization of Majorana fermions, which in turn are expected to play an important role in future fault-tolerant topological quantum computation^2^,^3^ due to their exotic non-Abelian braiding statistics^4^,^5^. Many different schemes have been proposed to realize TSCs, including odd-parity pairing copper oxide superconductors^6^,^7^, surface states of topological insulators^8^,^9^ or two-dimensional (2D) Rashba spin-orbit coupled semiconductors^10^ proximity coupled with an *s-*wave superconductor, and 1D ferromagnetic Shiba chain on top of a conventional superconductor with strong spin-orbit coupling (SOC)^11^. These different innovative proposals continue to stimulate active research efforts on definitive experimental realization of TSCs and observation of Majorana fermions.

In essentially all the compelling experimental demonstrations of TSCs and/or Majorana fermions reported so far^11^,^12^,^13^,^14^,^15^, the presence of certain types of disorder must be unavoidable. Indeed, the effects of random disorder on the properties of both conventional and unconventional superconductors have been extensively investigated^16^,^17^,^18^,^19^. Earlier studies had primarily evolved around disorder effects on the superconducting properties^16^. More recently, in 1D topological superconducting systems, a nontopological localized phase has been obtained when the strength of the introduced Anderson disorder is in the strong scattering regime^17^,^18^,^19^. In addition to these less desirable cases where the presence of disorder destroys the salient physical properties of the systems, there have also been numerous examples showing that properly introduced disorder can promote the emergence of intriguing phenomena in otherwise clean but ordinary host systems^20^,^21^,^22^,^23^,^24^. One such example is the recent discovery of the topological Anderson insulating (TAI) state in HgTe quantum wells^21^. It was shown that the presence of Anderson disorder can convert a topologically trivial insulator into a topologically nontrivial insulator (or the TAI state), with a quantized conductance of *G*_0_ = 2*e*^2^/*h*^22^,^23^,^24^. In view of the widespread presence of disorder in realistic systems and the recent developments surrounding the TAI, it is naturally intriguing to investigate the feasibilities of disorder-induced topological phase transitions in otherwise normal superconducting systems, especially the potential realization of disorder-assisted TSCs.

Here we carry out a comparative study using complementary theoretical approaches to explore the potential existence of topological phase transitions in 2D Rashba spin-orbit coupled superconductors by proper introduction of different types of disorder (see Fig. 1). First, we use the self-consistent Born approximation (SCBA) to investigate the disorder renormalized density of states (DOS) of the systems, and show that a topologically trivial superconductor can be driven into a chiral TSC upon diluted doping of isolated magnetic disorder. Secondly, whereas the superconducting nature of a topological superconductor is found to be robust against Anderson disorder, the topological nature is not, converting the system into a topologically trivial state even in the weak scattering limit. These topological phase transitions proceed via intricate narrowing-closing-reopening processes of the quasiparticle gap of the paired electrons in nontrivial manners, and are quantitatively characterized by the variations in the topological invariant. The central findings are also confirmed by solving the Bogoliubov-de Gennes (BdG) equations self-consistently within a tight-binding model. We discuss the validity of the present model studies in connection with existing experiments, and provide disorder-based new design schemes towards eventual materials realization of TSCs.

## Results

### Theoretical model

To start, we consider a 2D electron gas with a Rashba-type SOC and a Zeeman field *h*. Discussions on potential experimental realizations of such systems will be deferred to near the end of this paper. By introducing a proper attractive interaction between the electrons, the system will enter a superconducting state, and can further be classified as a topologically nontrivial superconductor when the Zeeman field exceeds a critical value *h*_*c*_^25^,^26^,^27^,^28^. The total Hamiltonian *H* of such a 2D system contains two parts. The first part is given as 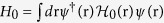, where 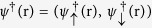 and 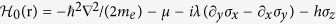. Here, *m*_*e*_ is the effective mass of the electrons, *μ* is the chemical potential, *λ* is the strength of the Rashba SOC, and *σ*_*i*_ (*i* = *x, y, z*) are the Pauli spin matrices. The second part is the on-site attractive interaction between the electrons, described by 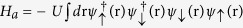, where *U* is the attraction strength. In the momentum space, by treating the two-body interaction *H*_*a*_ within the mean-field approximation, the total Hamiltonian *H* can be written as 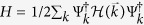, with


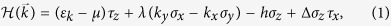


where 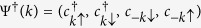 are the field operators in the Nambu spinor basis, 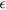
_*k*_ = *ħ*^2^*k*^2^/2*m*_*e*_, *τ*_*i*_ (*i* = *x, y, z*) are the Pauli matrices acting on the particle-hole degrees of freedom, and Δ = *U*∑_*k*_〈*c*_−*k*↓_*c*_*k*↑_〉 is the mean-field superconducting order parameter. The system described by Eq. (1) is a topologically nontrivial superconductor when 
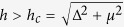
29,30. Here we also differentiate the present model systems, where time-reversal symmetry is explicitly broken by the Zeeman term, with those considered in some earlier studies in which time-reversal symmetry is protected^31^,^32^.

To describe the interactions between the electrons and disorder introduced into the system, we consider a local scattering Hamiltonian *H*_*in*_ = −∫*d*r*ψ*^†^(r)*V*_*in*_*δ*(r − r_0_)*ψ*(r), whose simple form is able to capture the essential physics to be exploited in the present study. In the case of magnetic disorder, we choose an isotropic form given by *V*_*in*_ = *J*(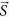
⋅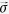), where *J* denotes the exchange coupling strength between the electrons and magnetic disorder, 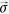 = (*σ*_*x*_, *σ*_*y*_, *σ*_*z*_) is the electron spin, and 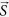 = (*S*_*x*_, *S*_*y*_, *S*_*z*_) is the moment of a magnetic dopant. In the following studies, we adopt a typical value of about 2.45 Bohr magnetons for all the magnetic dopants. For usual Anderson disorder, *V*_*in*_ is the on-site disorder potential *V* distributed uniformly in the interval (−*V*_0_, *V*_0_). In Nambu notations, the disorder scattering Hamiltonian can be rewritten as


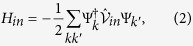


where 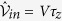 for the Anderson disorder and 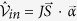 for the magnetic disorder. Here the effective electron spin operator 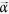 is defined as 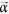 = 1/2[(1 + *τ*_*z*_)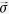 + (1 − *τ*_*z*_)*σ*_*z*_ 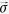 *σ*_*z*_]. Hamiltonian (2) is a general form describing the interaction between disorder and superconducting quasiparticles, and Eqs (1) and (2) provide the framework for further studying the disorder-induced effects on the topological phases of such spin-orbited coupled superconducting systems.

We adopt the Green’s function formalism to study the effects induced by multiple disorder in a superconductor. After averaging over the randomly distributed disorder, the Matsubara Green’s function of the system described by Eqs (1) and (2) is given as


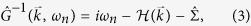


where *ω*_*n*_ = (2*n* + 1)*πk*_*B*_*T, k*_*B*_ is the Boltzmann constant, *T* represents the temperature, and 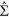 is the self-energy. In the presence of disorder, the superconducting order parameter is determined by the self-consistent equation


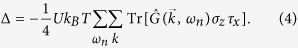


And within the SCBA, 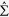 is given by


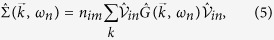


where *n*_*im*_ is the concentration of the disorder. Taking into account of the symmetry restriction and the matrix structure of 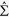, the self-energy effects can be manifested as disorder-induced renormalizations of *ω*_*n*_, *μ, h*, and Δ. As shown in Eq. (5), 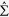 is independent of the momentum, leading to a renormalized form of the Green’s function as 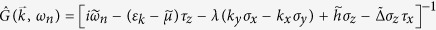, obtained with the replacements of 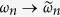, 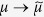, 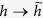, and 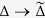. The solution of Eq. (5) gives rise to a set of self-consistent equations


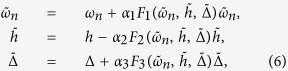


where 
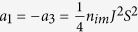
, 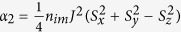 for the magnetic disorder, and 
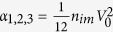
 for the Anderson disorder. The detailed analytical formulas for 
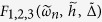
 are given in the method part. Here we also ignore the renormalization of 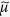 as it simply changes the chemical potential, which can be preset suitably, unlike the TAI cases, where the renormalization of 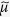 has significant effects^22^,^23^,^24^. The disorder-averaged Green’s function 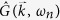 can then be obtained by solving the self-consistent equations of Eqs (6) and (4) simultaneously.

### Topological phase transitions

To quantitatively characterize the disorder effects, we investigate the DOS defined by 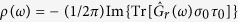, where 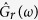 is the retarded Green’s function obtained by performing analytical continuation on 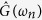. It is well known that a single magnetic impurity can lead to the appearance of the Yu-Shiba states inside the superconducting gap^33^,^34^, and multiple magnetic impurities can also cause sizable renormalization of the superconducting gap even upon diluted doping^16^. In contrast, according to Anderson’s theorem^35^, a conventional *s-*wave superconductor can be barely influenced by potential disorder. However, recent studies show that a nonmagnetic potential disorder can also be able to induce mid-gap bound states in a TSC^36^,^37^. The present study extends these earlier understandings to topological aspects of such spin-orbit coupled superconducting systems in the presence of multiple disorders.

**Figure 2:**
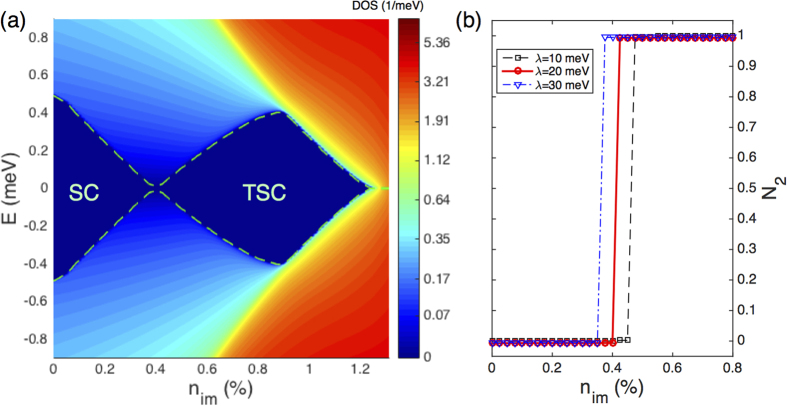
Topological phase transitions induced by the magnetic disorder, with the initial superconducting state to be topologically trivial. (**a**) The DOS as a function of *n*_*im*_ with *J* = 100 meV. The dashed (green) curves highlight the edges of the quasiparticle gap. (**b**) Topological invariant as a function of *n*_*im*_ and the Rasgba SOC strength *λ*, with the solid (red) curve corresponding to the transition shown in (**a**). Other parameters include 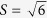, *m*_*e*_ = 0.01 meV⋅Å^−2^, *h* = 2.5 meV, and *U* = 160 meV, leading to Δ ≈ 3 meV.

Here we first study the effects induced by the magnetic disorder. Bases on the study of DOS, Fig. 2(a) highlight the most striking finding of the present study, namely, an initial topologically trivial superconductor can be driven into a TSC via diluted doping of randomly distributed magnetic disorder. The quasiparticle of the system undergoes a narrowing, closing, and reopening process upon increasing the concentration *n*_*im*_ of the magnetic disorder. A critical concentration *n*_*c*_ for such a topological quantum phase transition is clearly identifiable. Another feature of Fig. 2(a) is that the quasiparticle gap closes again upon further increasing *n*_*im*_. In this regime containing sufficient magnetic impurities (with *n*_*im*_ > 1.2% in the present study), the system is a gapless superconductor with non-vanishing superconducting order parameter^16^. However, below this concentration regime, we do have a sizable range of *n*_*im*_ that supports the existence of TSCs with the other important parameters chosen from physically realistic ranges.

**Figure 3:**
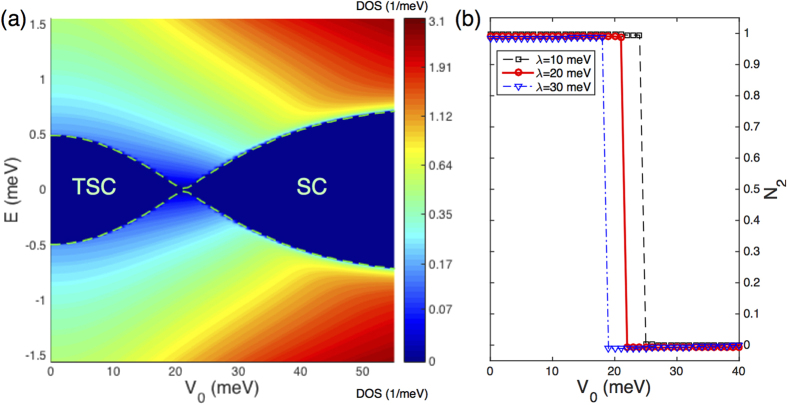
Topological phase transitions induced by the Anderson disorder, with an initial topological superconducting state. (**a**) The DOS as a function of the disorder strength *V*_0_. The dashed (green) curves highlight the edges of the quasiparticle gap. (**b**) Topological invariant as a function of *V*_0_ and the Rasgba SOC strength *λ*, with the solid (red) curve corresponding to the phase transition shown in (**a**). Other parameters include *m*_*e*_ = 0.01 meV⋅Å^−2^, and *U* = 160 meV, *h* = 3.5 meV, leading to Δ ≈ 3 meV.

Once a TSC is achieved, it is intriguing to examine how robust the system is in the presence of other types of disorder. The most common type of disorder is the Anderson disorder, whose effects on both the topological and superconducting aspects of a TSC are investigated next. As shown in Fig. 3(a), we find a topological phase transition from the TSC state to a trivial superconducting state. Qualitatively, an increase in the strength *V*_0_ of the on-site Anderson disorder will gradually close the quasiparticle gap of the TSC. When *V*_0_ crosses a critical value *V*_*c*_, the quasiparticle gap reopens, and the system enters a trivial superconducting state. These findings suggest that, as far as Anderson disorder is concerned, a cleaner superconducting system is better for the realization of TSC. We also note that further increase of *V*_0_ to the strong scattering regime is likely to transform the system into the Anderson localized state^17^,^18^,^19^.

To measure the occurrence of these topological phase transitions induced by disorder, we further calculate the topological invariant for various parameter regions. Due to the presence of the randomly distributed disorder, their interaction with the host system makes it impossible to calculate the Chern number using the standard Berry phase approach based on a pure band picture^38^. Here we adopt an alternative and more general formula for evaluating the topological invariant, which relies on the full Green’s function of the interacting system as^39^,^40^,^41^:


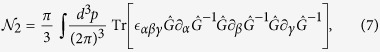


where 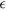
_*αβγ*_ is the Levi-Civita symbol, 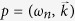, 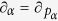 acting on the immediately following 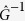, and the summations over *α, β, γ* are implied. Here we note that 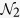 is the TKNN integer of a 2D system. The full Green’s function of such a disordered system can be obtained self-consistently, and the topological invariant can then be straightforwardly calculated using Eq. (7). As shown in Fig. 2(b), the topological invariants jump from 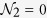 to 1 at the critical values of *n*_*im*_, which confirms that a topologically trivial SC (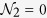) can be driven into a chiral TSC (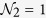) via increasing magnetic doping. After the realization of TSC, Fig. 3(b) illustrates the topological phase transition from a TSC to trivial SC induced by increasing the *V*_0_ of the Anderson disorder. In addition, we also find that the enhancement of the SOC strength will promote these topological phase transitions in both cases of the magnetic and Anderson disorder. Collectively, these results offer strong evidence for the existence of rich topological phases induced by proper choices of multiple disorders.

### Numerical BdG solutions

In this section, we numerically investigate the disorder-induced effects within a corresponding tight-binding model of the system described by Eq. (1), which also enable us to gain further insights on the spatial distributions and in particular inhomogeneities in the superconducting and topological phases. By projecting Eq. (1) on a 2D square lattice, we have


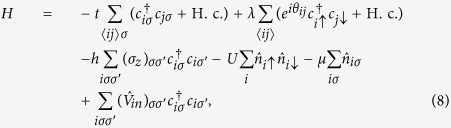


where *t* is the nearest-neighbor hopping term, 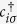 and *c*_*iσ*_ are the creation and destruction operators for an electron with spin *σ* on site r_*i*_ of the square lattice, 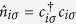 is the particle number operator, and *θ*_*ij*_ is the angle between (r_*j*_ − r_*i*_) and the *x* axis. Here, we solve the disordered system characterized by Eq. (8) within the standard mean-field BdG approach, 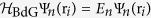, where


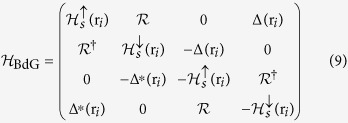


is the BdG Hamiltonian, *E*_*n*_ is the eigenenergy of the corresponding quasiparticle wave function 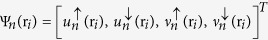 defined in the Nambu spinor space. Here 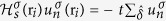

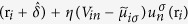
, 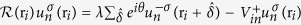, 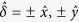, Δ(r_*i*_) is the local superconductor order parameter, and 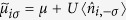 is the modified chemical potential. We note that 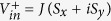 for the magnetic disorder and vanishes for the Anderson disorder. Starting with some initial guess values of Δ(r_*i*_), we first numerically solve the BdG Hamiltonian on a *N* × *N* square lattice with periodic boundary conditions. Next, we calculate the local pairing amplitudes and particle density given by


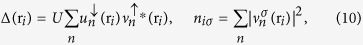


and iterate this process until self-consistent values of *n*_*iσ*_ and Δ(r_*i*_) at each site are achieved.

**Figure 4:**
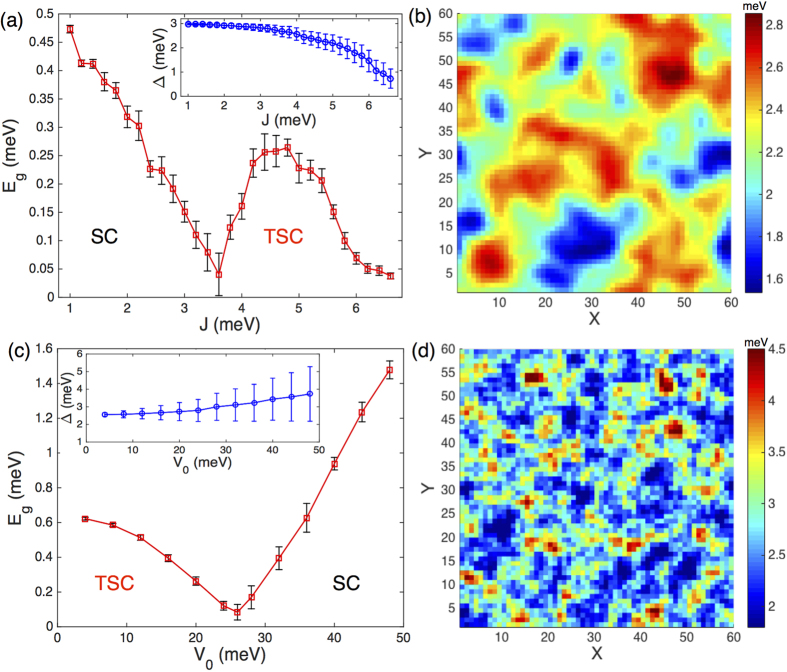
The upper and lower panels show the numerical results of a 60 × 60 square lattice in the presence of on-site Anderson and magnetic disorder, respectively. (**a**) Quasiparticle gap as a function of the on-site magnetic coupling strength *J*. (**b**) The spatial variations of ∆(r_i_) for randomly distributed magnetic disorder with *J* = 4.8 meV. (**c**) Quasiparticle gap as a function of the disorder strength V_0_. (**d**) The spatial variations of ∆(r_i_) for the Anderson disorder with V_0_ = 10 meV. The error bars in (**a**) and (**c**) show the standard deviations of the mean for 10 samples, and the inserts give the averaged local superconducting order parameters. Other parameters include 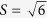, *U* = 160 meV, and *t* = 50 meV, corresponding to *m*_*e*_ = 0.01 meV⋅Å^−2^.

Numerical solutions of the BdG equation can yield the whole quasiparticle spectrum in the presence of the disorder. As shown in Fig. 4(a) and (c), there exists two regimes of the disorder strength showing nonzero quasiparticle gaps. Similar to the SCBA results, we also observe gap narrowing, closing, and reopening processes by increasing the strength of both the magnetic and Anderson disorder. These behaviors are qualitatively the same as that shown in Figs 2(a) and 3(a). We note that, in the numerical calculations, the magnetic disorder is treated as on-site spin with randomly oriented directions for computational convenience. From the quantitative perspective, as shown in Fig. 2(a), the critical concentration characterizing the topological phase transition is *n*_*c*_ = 0.4%, translating into *J* = 3.2 meV for on-site disorder, which is comparable to the critical coupling strength *J*_*c*_ = 3.6 meV illustrated in Fig. 4(c). For the Anderson disorder, the critical value of *V*_0_ obtained here is about 26 meV, which is also comparable to *V*_*c*_ = 22 meV shown in Fig. 3(a). The agreements between the results of the numerical simulations and the earlier analytical solutions within the SCBA stem from the fact that both the magnetic and Anderson disordered systems are within the weak-scattering regime.

Based on these qualitative and quantitative comparisons between the results obtained analytically earlier and numerically here, we can conclude affirmatively that rich topological phases can indeed be induced by properly introducing disorder. In particular, a topologically trivial SC can be readily driven into a TSC upon diluted doping of independent magnetic impurities, and a topological phase transition from TSC to SC will be induced by Anderson disorder. In Fig. 4(b), we also plot the spatial variations of Δ(r_*i*_) in the presence of magnetic disorder. It is natural to expect some regions to be TSC while others are topologically trivial SC. Therefore, the boundaries separating topologically distinct regions may offers a promising platform for observing and detecting Majorana fermions. In the present study, the Majorana edge states mix with the bulk states due to quantum size effects, making it difficult to distinguish them from the total energy spectrum. However, such a difficulty may be overcome naturally in realistic superconducting systems with much larger topologically distinct regions.

Before closing, we assess the validity of the present model study by briefly discussing candidate systems for potential experimental observation of such disorder-induced topological phase transitions. We could consider a superconductor thin film with strong SOC, such as Pb^11^,^42^,^43^,^44^ or PbBi alloyed films grown on semiconducting substrates. In particular, signatures of disorder effects on the superconducting gap have been observed in a very recent study, which has been attributed to the the effects of strong Rashba SOC in such systems due to the lacking of the inversion symmetry caused by the substrates^44^. Therefore, by further doping magnetic elements into or on the surface of such 2D superconductors, those topologically trivial systems may be converted into TSCs. Our results may also be observed in the recently realized spin-orbit coupled 2D ultracold atomic Fermi gases of ^40^K^45^ and ^6^Li atoms^46^, which are shown to be topological superfluids. Finally, the present findings can be naturally extended to 1D and 3D cases.

## Discussion

In summary, we have theoretically explored the feasibilities of altering the topological properties of a 2D Rashba spin-orbit coupled superconductors by proper introduction of magnetic or Anderson disorder. We found that a topologically trivial SC can be driven into a nontrivial chiral TSC upon diluted doping of isolated magnetic disorder, which induces an intricate narrowing, closing, and reopening of the quasiparticle gap. Furthermore, whereas the superconducting nature of a TSC is found to be robust against Anderson disorder, the topological nature is not, converting the system into a topologically trivial state even in the weak scattering limit. The validity of the present model study has been discussed in connection with existing experiments. Collectively, the central findings presented here provide disorder-based new design schemes towards eventual materials realization of TSCs, which in turn may find important applications in future quantum computation devices.

## Method

### The derivation of self-consistent equations

In this part, we focus on the details of the analytical derivations of 
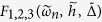
 defined in Eq. (6). Taking into account of the symmetry restriction and the matrix structure of 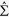, a set of self-consistent equations can be obtained as


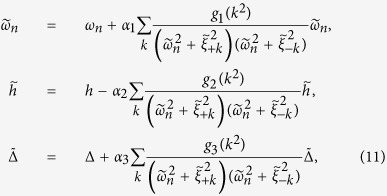


where *α*_1,2,3_ have been defined in the main text, and


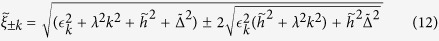


are the disorder-averaged energy spectrum of the superconducting quasiparticle. The defined functions *g*_1,2,3_(*x*) have the following form


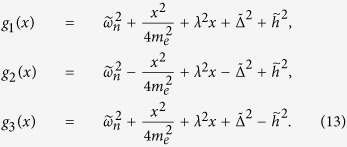


Further simplification of Eq. (11) can be reached by performing the integration over the wave vector 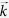, and defining 
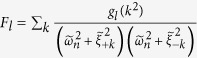
, where *l* = 1, 2, 3. *F*_*l*_ can be rewritten as


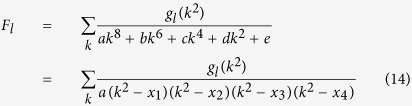


where


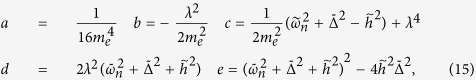


and


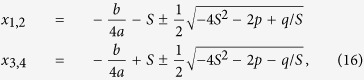


by replacing the corresponding notations with


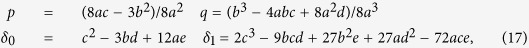


and


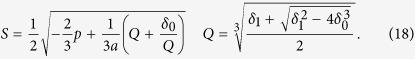


Consider the following Fourier transformation


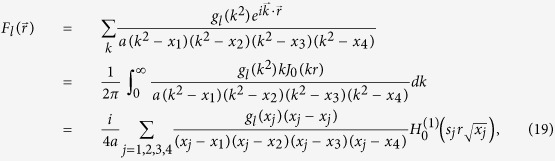


where *J*_0_(*x*) is the Bessel function of the first kind, 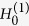 is the Hankel function, and 
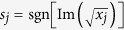
 guarantees the contour integrations in Eq. (19) are performed in the up-half complex plane.

Taking r → 0 we thus find the following asymptotic forms of *F*_*l*_


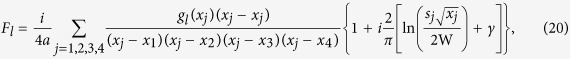


where W is a large band cutoff, *γ* is the Euler constant, *F*_*l*_ is a function of 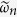, 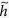, and 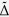.

## Additional Information

**How to cite this article**: Qin, W. *et al*. Disorder-induced topological phase transitions in two-dimensional spin-orbit coupled superconductors. *Sci. Rep.* **6**, 39188; doi: 10.1038/srep39188 (2016).

**Publisher's note:** Springer Nature remains neutral with regard to jurisdictional claims in published maps and institutional affiliations.

## Figures and Tables

**Figure 1:**
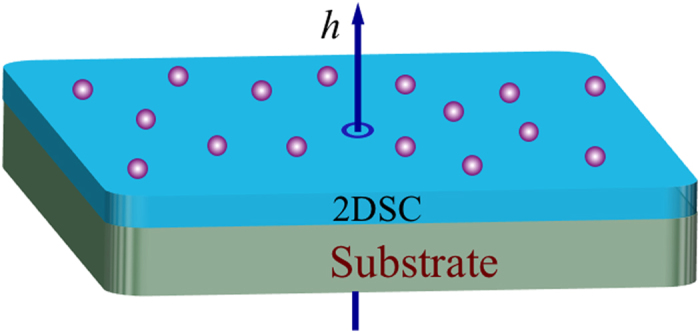
Schematic of randomly distributed disorder on a 2D superconductor (2DSC) with spin-orbit coupling and the presence of a Zeeman field *h*.
